# Case report: Clinical and genetic characterization of a novel *ALDH7A1* variant causing pyridoxine-dependent epilepsy, developmental delay, and intellectual disability in two siblings

**DOI:** 10.3389/fpsyt.2024.1501238

**Published:** 2024-12-23

**Authors:** Mustafa A. Salih, Albandary AlBakheet, Rawan Almass, Ahlam A. A. Hamed, Ali AlOdaib, Namik Kaya

**Affiliations:** ^1^ Consultant Pediatric Neurologist, Health Sector, King Abdulaziz City for Science and Technology, Riyadh, Saudi Arabia; ^2^ Translational Genomic Department, Center for Genomic Medicine, King Faisal Specialist Hospital and Research Centre, Riyadh, Saudi Arabia; ^3^ Medical Genetics Department, Center for Genomic Medicine, King Faisal Specialist Hospital and Research Centre, Riyadh, Saudi Arabia; ^4^ Consultant Pediatric Neurologist, Faculty of Medicine, University of Khartoum, Khartoum, Sudan; ^5^ Research and Innovation, King Faisal Specialist Hospital and Research Centre, Riyadh, Saudi Arabia

**Keywords:** ALDH7A1, novel variant, pyridoxine-dependent epilepsy, intellectual disability, neonatal seizures

## Abstract

**Background:**

Pathogenic variants in *ALDH7A1* are associated with pyridoxine-dependent epilepsy (PDE), a rare autosomal recessive disorder characterized by epileptic seizures, unresponsiveness to standard antiseizure medications (ASM), and a response only to pyridoxine. Here, we report two patients (from a consanguineous family) with neonatal seizures and developmental delay.

**Case presentation:**

Patient 1 (a 13-year-old girl) was born normally at term. Her pregnancy was complicated by antiphospholipid syndrome, and persistent vomiting was managed with several medications, including pyridoxine (40 mg daily). Seizures occurred 6 h after birth and did not respond to antiseizure medications. However, they ceased 2 days later when pyridoxine (40 mg daily) was administered. She continued her medications and had delayed early milestones. Phenobarbitone was discontinued at 18 months, and pyridoxine was increased to 100 mg daily at 8 years of age. She was able to join a regular school and performed well. Patient 2, a 12-year-old boy, was delivered normally at term. Seizures started 10 h after birth, and he immediately received 40 mg of pyridoxine. Seizures have been controlled since then, and he experienced delayed milestones. Pyridoxine was increased to 100 mg daily at 7 years of age. He is currently in fifth grade and has dyslexia. Whole exome sequencing (WES) revealed that both patients 1 and 2 harbor a novel homozygous missense variant in *ALDH7A1* (NM_001202404: exon 12: c.1168G>C; (p.Gly390Arg)).

**Conclusion:**

The present study reports a novel *ALDH7A1* variant causing PDE and highlights the associated developmental delay and intellectual disability, despite early seizure control treatment.

## 1 Introduction

Variants in the *ALDH7A1* gene are associated with pyridoxine-dependent epilepsy (PDE), a rare autosomal recessive disorder characterized by etiology-specific developmental and epileptic seizures specific to the condition. These seizures typically occur in the first hours of life, are unresponsive to standard antiseizure medications (ASM), and respond only to pyridoxine administration (1). The incidence of PDE ranges between 1:65,000 and 250,000 live births (2, 3). Timely diagnosis is imperative to prevent uncontrolled seizures, which may jeopardize neurocognitive development (4). Atypical presentations are known to occur, and seizures may manifest after the neonatal period (5). In some patients, seizures may initially respond to standard ASM (6). Global developmental delay and/or intellectual disability have been recorded in 75% of pyridoxine-dependent epilepsy patients, even with early treatment initiation and seizure control (7–9).

The *ALDH7A1* gene, also known as aldehyde dehydrogenase 7 family member A1, encodes an enzyme within the aldehyde dehydrogenase (ALDH) family. ALDH enzymes are essential for metabolizing aldehydes, toxic byproducts produced during various cellular processes, such as alcohol metabolism and lipid peroxidation. *ALDH7A1* specifically catalyzes the conversion of these aldehydes into less harmful carboxylic acids. Specifically, it acts on a variety of aldehydes, including acetaldehyde and 4-hydroxybutyric acid. *ALDH7A1* is involved in several metabolic pathways, notably the breakdown of lysine, an essential amino acid, in the mitochondrial matrix. In detoxification, *ALDH7A1* helps prevent the accumulation of toxic aldehydes, protecting cells from potential damage. A deficiency in *ALDH7A1* can also lead to hyperammonemia due to lysine accumulation. The enzyme is expressed in various tissues, including the liver, brain, and kidney. One or more of the accumulated biomarkers are thought to contribute to the developmental delay and/or intellectual disability observed in patients who are treated late, and lysine reduction therapies (LRT) have been implemented to reduce these consequences (10).

Here, we investigate a novel pathogenic homozygous variant in *ALDH7A1*, primarily causing neonatal seizures and developmental delay in two patients from a consanguineous Sudanese family living in Saudi Arabia. The older patient (13-year-old girl) exhibited delayed early milestones but was able to attend regular school, performing well in the seventh and eighth grades. The younger patient had similar features and had dyslexia in both Arabic (his native language) and English.

## 2 Methods

### 2.1 Sample collection

Two patients from a consanguineous Sudanese family residing in Saudi Arabia were recruited with approval from the Institution’s Research Advisory Council (Ethics Statement) at King Faisal Specialist Hospital and Research Center, Riyadh, KSA (KFSHRC, IRB-approved protocols, RAC#2180004). The participants signed written informed consent forms.

### 2.2 Molecular genetic detection

Peripheral blood samples (5 ml) were collected in EDTA tubes from both affected and unaffected individuals within the participating family. DNA was isolated using the Gentra^®^ Puregene DNA Purification Kit (Gentra Systems Inc., Minneapolis, MN, USA). PCR was performed with primers designed by Primer3, followed by direct sequencing on an ABI PRISM 3100 Genetic Analyzer (Applied Biosystems, Foster City, CA, USA), in accordance with the manufacturer’s instructions. WES was conducted on the patient’s DNA, and variants were filtered and analyzed for *in silico* pathogenicity as previously described (11, 12). DNA samples were genotyped using Affymetrix Axiom chips, following the manufacturer’s protocols (Affymetrix Inc., Santa Clara, CA, USA). The analysis was performed as previously described (11). Autozygome coordinates were considered during the WES variant filtering process.

## 3 Results

### 3.1 Clinical features

#### 3.1.1 Patient 1 (IV:6)

**Figure 1:**
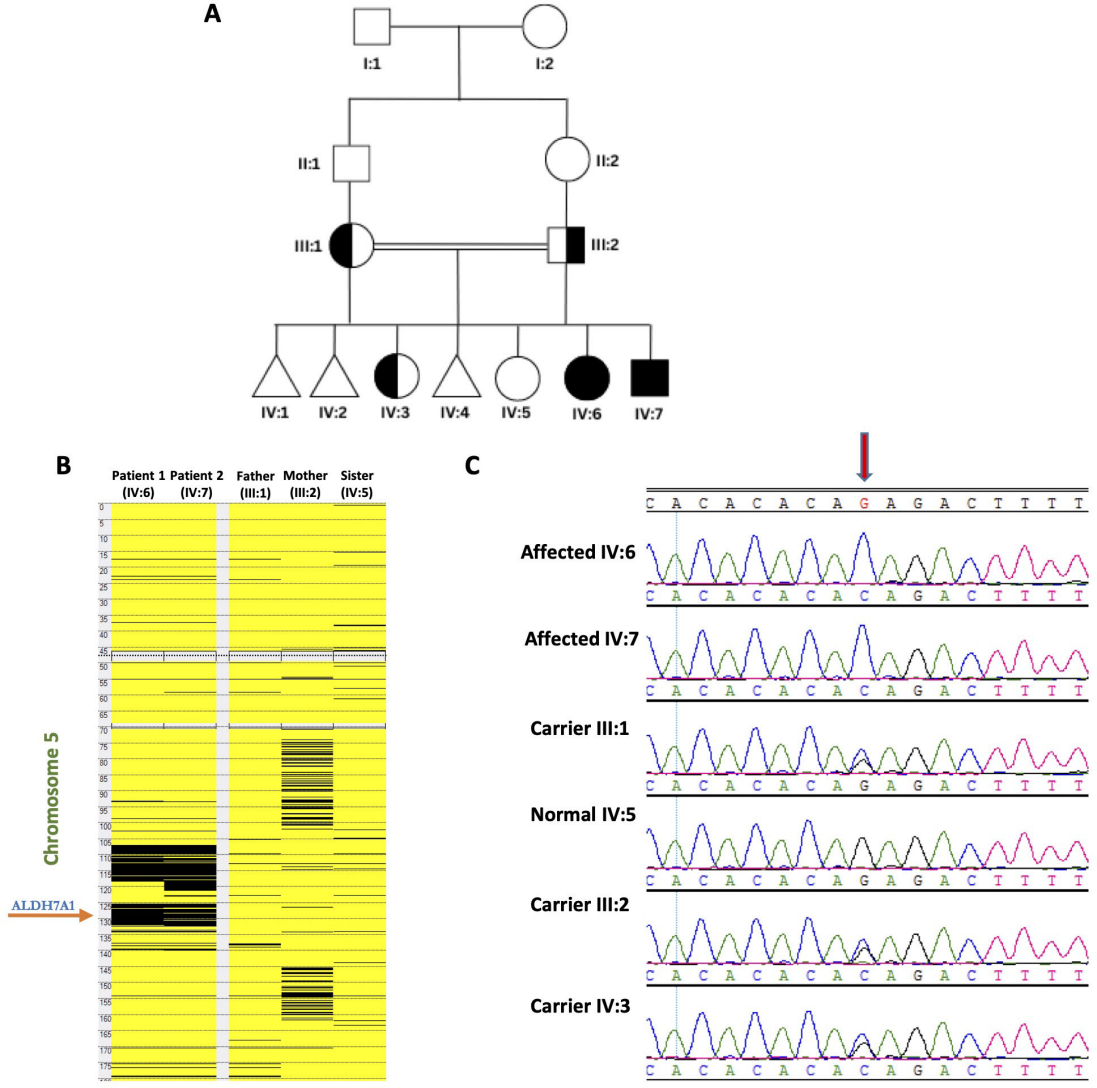
Genetic analyses of the families: **(A)** Pedigree of the studied family showing two affected members. **(B)** Homozygosity mapping indicating the location of the *ALDH7A1* gene within the major ROH in the family. **(C)** The chromatograms from Sanger Sequencing revealing segregation of the mutation in the family members (The red arrow highlights the position of the mutation).

Patient 1 is a 13-year-old girl, born as the third child to healthy, first-degree consanguineous Sudanese parents living in Saudi Arabia (**Figure 1**). She was delivered at 38 weeks via normal spontaneous vaginal delivery after a smooth course of labor. Her Apgar score was 8 at 5 min. She received naloxone due to pethidine administered to her mother and was admitted to the Neonatal Intensive Care Unit (NICU) for pethidine depression. The pregnancy was complicated by antiphospholipid syndrome, for which the mother received 81 mg of aspirin tablets daily and 40 mg of clexane subcutaneously daily until term. The mother experienced persistent vomiting, which subsided only after delivery despite attempts with metoclopramide (Primperan), promethazine (Phenergan), pyridoxine (40 mg daily), and ondansetron during pregnancy. At 34 weeks, the mother had pre-term contractions and a suspected urinary tract infection, which was treated with cephradine, ritodrine (Yutopar), and a course of dexamethasone injections. The mother had three previous miscarriages. Six hours after birth, patient 1 started to have seizures that did not respond to phenobarbitone and midazolam, and she was ultimately ventilated due to recurrent seizures. Two days later, pyridoxine (40 mg daily) was administered via nasogastric tube, after which no further seizures were recorded. She was extubated on day 5 and continued on phenobarbitone, midazolam, pyridoxine, and empirical antibiotics.

Complete blood count, biochemistry, arterial blood gas, blood culture and sensitivity, lumbar culture results, coagulation screen, and tandem mass spectrometry were normal. Electroencephalography (EEG) showed epileptiform discharges while staying in the NICU. One month after discharge, no epileptiform discharges were seen, but diffuse slowing was observed. Since then, and until now, repeated EEG examinations have been normal. Cranial magnetic resonance imaging (MRI), done for suspected cavernous sinus thrombosis on day 5, revealed normal findings. Pyridoxine metabolite tests (alpha-aminoadipic semialdehyde) were not done, as they were not accessible. She had delayed early milestones: started sitting at 14 months, crawling at 20 months, walking at 26 months, and spoke one word by 24 months. Phenobarbitone was stopped at the age of 18 months, and the dose of pyridoxine was increased to 100 mg daily at the age of 8 years following confirmatory genetic results. This was adjusted to 300 mg daily at the age of 13 years. She managed to join a regular school, performing well in seventh and eighth grades.

#### 3.1.2 Patient 2 (IV:7)

Patient 2, the younger brother of patient 1, is currently aged 12 years old (**Figure 1**). He was delivered at term via normal spontaneous vaginal delivery. Seizures started 10 h after birth, and he immediately received 40 mg of pyridoxine. Since then, the seizures have been controlled. Electroencephalography performed 2 days later revealed no abnormalities, and magnetic resonance imaging (done at the age of 1 month) was normal. Pyridoxine metabolite tests (alpha-aminoadipic semialdehyde) were not performed, as they were not accessible. He had delayed milestones, walked at 18 months, and was able to make sentences by 3 years of age. The dose of pyridoxine was increased to 100 mg daily at the age of 7 years following confirmatory genetic results. This was adjusted to 300 mg daily at the age of 12 years. He is currently in fifth grade and has dyslexia in both Arabic (his native language) and English.

### 3.2 Molecular genetic results

WES analysis identified a novel homozygous variant in *ALDH7A1* (NM_001202404: exon 12: c.1168G>C; p.Gly390Arg) as the only strong candidate underlying the disorder. The gene is in one of the major runs of homozygosity (ROH) block detected on chromosome 5q31 (**Figure 1**). Confirmatory Sanger sequencing demonstrated complete segregation of this variant within the family (**Figure 1**). Based on this, an elder sister (IV:3) was found to be a heterozygous carrier and is clinically normal. The c.1168G>C variant causes a missense change involving the alteration of a conserved nucleotide. The variant was absent in control chromosomes in the GnomAD project, as analyzed by MutationTaster and Genebe. According to the American College of Medical Genetics (ACMG), the c.1168G>C variant is classified as likely pathogenic based on the following criteria: scores: 11, CADD: pathogenic (31), PolyPhen: damaging (D), PM1: moderate, PM2: moderate, and PP3: strong. No clinical diagnostic laboratories have submitted clinical-significance assessments for this variant to ClinVar.

## 4 Discussion

The present study highlights a rare, treatable genetic disorder associated with intellectual disability, namely PDE, where timely diagnosis is imperative. It describes the clinical phenotype of a novel familial *ALDH7A1* mutation causing neonatal seizures and developmental delay in two patients from a consanguineous family. Both patients manifested the classic presentation of ALDH7A1-related pyridoxine-dependent epilepsy, with seizures starting shortly after birth (13). Patient 1 did not respond to antiseizure medications and was ultimately placed on a ventilator due to recurrent seizures. She responded to pyridoxine (40 mg daily), administered 2 days later via nasogastric tube, after which no further seizures were recorded. Seizures started 10 h after birth in the younger brother (patient 2), who immediately received 40 mg of pyridoxine. No further seizures occurred since then. Following confirmatory genetic results, pyridoxine was increased to 100 mg daily in both patients. However, this is lower than the current recommended dose for children, adolescents, and adults (range 5–30 mg/kg/day), along with dietary modifications aimed at reducing lysine intake (4, 13). Pyridoxine was recently adjusted for both patients to comply with the recommended dose. An EEG performed on patient 1 during their stay in NICU showed epileptiform discharges. No epileptiform discharges were seen 1 month after discharge, but diffuse slowing was noted, and repeated EEG examinations have been normal. EEG recordings are known to be variable and nonspecific in pyridoxine-dependent epilepsy caused by *ALDH7A1* (4, 14). Reported abnormalities include high-voltage delta waves, burst-suppression patterns, and hypsarrhythmia (6, 15, 16). On the other hand, a cranial MRI performed on day 5 for patient 1 revealed normal findings. Reported MRI abnormalities in ALDH7A1-associated pyridoxine-dependent epilepsy include hypoplasia of corpus callosum, cortical dysplasia, mega cisterna magna, cerebellar hypoplasia, ventriculomegaly, and hydrocephalus (13, 17–19).

Intellectual disability, primarily affecting expressive language, is common in this disease, with a higher risk correlated with the length of the delay in diagnosis and initiation of appropriate pyridoxine therapy (7, 13, 20). The neurodevelopmental disability, affecting approximately 75%, ranges from mild to severe intellectual disability and includes autistic features (4, 7, 21, 22). The longer the time between the initial administration of antiseizure medication, before suspecting PDE, and the subsequent administration of pyridoxine, the worse the neurological outcome appears to be at follow-up (23). To avoid missing cases, a trial of pyridoxine as a first-line treatment in all patients with seizures of unknown etiology has been suggested (24). The availability of newborn screening and rapid molecular genetic testing are expected to improve the neurological outcome in ALDH7A1-associated pyridoxine-dependent epilepsy (4). Deficiency of alpha-aminoadipic semialdehyde in PDE results in impaired lysine degradation due to deficiency of alpha-aminoadipic semialdehyde dehydrogenase. This leads to the accumulation of toxic metabolites from impaired lysine metabolism (22). To reduce the accumulation of these neurotoxic metabolites, a lysine-restricted diet and pharmacologic doses of arginine, which acts as a competitive inhibitor of lysine transport, are currently employed in addition to pyridoxine for the management of PDE (25). A significant increase in developmental testing scores was reported with pyridoxine and LRT treatment provided during the first 6 months of life (25). It is noteworthy that patient 1 received 40 mg of pyridoxine daily prenatally, which was administered to her mother due to persistent vomiting. This might have influenced the neurological outcome (16), as, despite early delayed development, she was able to join a regular school with good performance. Her younger brother, who also received 40 mg of pyridoxine immediately after his first seizure, also experienced delayed early development. He was also able to join a regular school but has dyslexia. The influence of genotype might explain the relatively favorable outcome in these siblings (26).

We identified a novel pathogenic homozygous variant in *ALDH7A1* (c.1168G>C) in two patients, which has not been previously reported. This variant affects a highly conserved amino acid position, potentially impacting protein functionality. Bioinformatic prediction tools suggest the variant’s pathogenicity. The parents of these siblings also carry the same mutation. Collectively, this evidence supports the interpretation that this variant is disease-causing. To date, a total of 143 variants have been reported for *ALDH7A1*, with most being missense/nonsense (*n* = 82), along with 26 splicing, 14 small deletions, seven small insertions, two small indels, 21 gross deletions, and one gross insertion. Fang et al. (26) explored the genotype–phenotype associations concerning variant site, type, and zygosity. The findings showed that patients with homozygous L455P mutations were more likely to experience clonic seizures, while those with homozygous E427Q mutations had a higher likelihood of developing ventriculomegaly, cysts, and abnormal white matter signals (26). Furthermore, patients with two in-frame deletion variants were more prone to myoclonic seizures, whereas those with homozygous variants were more likely to manifest a severe phenotypic abnormality including early-neonatal seizures, status epilepticus, and epileptic encephalopathy (26).

Identifying mutations in *ALDH7A1* through genetic testing confirms the diagnosis of PDE, allowing for the timely initiation of pyridoxine therapy, which is critical for preventing ongoing seizures and associated neurological damage. Moreover, genetic testing helps distinguish PDE from other forms of epilepsy, guides treatment decisions, and provides important information for family planning and genetic counseling. Understanding the role of *ALDH7A1* in PDE has been essential for diagnosing and managing this condition, emphasizing the importance of genetic testing in patients with unexplained epilepsy (23).

## 5 Conclusions

In conclusion, we reported a novel homozygous variant in the *ALDH7A1* mutation causing neonatal seizures and developmental delay in two patients from a consanguineous family. We also highlighted the associated developmental delay and intellectual disability despite early treatment and seizure control.

## Data Availability

The original contributions presented in the study are included in the article/supplementary material. Further inquiries can be directed to the corresponding author.
